# Developing treatment strategies for rare cancers

**DOI:** 10.18632/oncotarget.334

**Published:** 2011-09-27

**Authors:** Jennifer M. Atkinson, Richard J. Gilbertson

**Affiliations:** Departments of Developmental Neurobiology and Oncology, St Jude Children's Research Hospital, 262 Danny Thomas Place, Memphis, TN 38105

Trial therapies for rare cancers are usually selected empirically and often fail during clinical testing. The poor efficiency and limited success of this process is hampered by a lack of accurate models with which to perform pre-clinical studies, and small patient numbers that are likely to include distinct subtypes. These challenges are further compounded by increasing evidence that many cancers are heterogeneous diseases, composed of numerous different subtypes that are likely to require different therapies [1, 2]. Using a recently developed accurate model of a specific subtype of ependymoma, we have developed an approach to advance the efficiency and speed with which treatments for rarer cancers may be discovered, developed and prioritized [3].

Ependymomas are rare tumors of the brain and spinal cord that are incurable in up to 40% of cases. Like many human cancers, previous studies have indicated that ependymoma is a composed of different subtypes [1, 2]. To develop rational treatments of ependymoma, we are generating mouse models of each subtype in order to model human cancer heterogeneity during preclinical drug development [1]. Our first model faithfully recapitulates the histology and transcriptome of one form of human cerebral ependymoma (subtype-D): making it an attractive tool for developing treatments for this specific group of patients as well as understanding the signaling pathways important in sustaining the disease [1]. Further, since our models are generated from specific types of neural stem cells (NSCs), we are also able to test if candidate therapies are toxic to closely related normal cell types. Pre-clinical tools that allow measurement of drug efficacy as well as toxicity against stem and progenitor cells are especially important for developing treatments of children with cancer whose organ systems are immature [1].

Our report in the September 2011 issue of *Cancer Cell* describes an integrated, multiplatform in vitro and in vivo high throughput screen (HTS) of drug efficacy against our mouse model of subtype-D ependymoma, and concurrent assessment of toxicity against the parental normal embryonic cerebral NSC. This comprehensive approach identified several new candidate therapies for clinical development including tumor selective agents, as well as novel insights into the biology of ependymoma [3]. Through a combination of growth inhibition and kinome-wide binding assays, our studies demonstrated that inhibitors of the Insulin Growth Factor receptor signal pathway and centrosome cycle e.g., inhibitors of PLK1, are highly potent against subtype-D ependymoma. These kinases have not been previously implicated in the biology of ependymoma.

In addition to identifying kinases important for maintaining ependymoma, our study emphasizes the value of HTS for rationally repurposing existing anti-cancer drugs. Our HTS identified 5-Fluorouracil (5-FU) and two closely related compounds as highly selective and potent inhibitors of subtype-D ependymoma. Moreover, this potency and selectivity was confirmed *in vivo*. 5-FU has never been tested formally in patients with ependymoma. Indeed it is most unlikely that an early generation chemotherapeutic like 5-FU would ever be selected empirically for clinical trial. Consequently, we are now developing a clinical trial of 5-FU among children with ependymoma.

We are currently using the same methodology employed to produce subtype-D tumors to generate mouse models of the remaining eight ependymoma subtypes. Together with our integrated in vitro and in vivo screening approach, this battery of models should allow us to complete in months, numerous single and multidrug preclinical trials that would take decades to conduct in the clinic. Preclinical strategies such as these hold significant promise to identify and prioritize therapies for rational clinical trials among patients with specific subtypes of cancer.
